# Experimental studies of vibrational modes in a two-dimensional amorphous solid

**DOI:** 10.1038/s41467-017-00106-5

**Published:** 2017-07-10

**Authors:** Ling Zhang, Jie Zheng, Yinqiao Wang, Lei Zhang, Zhaohui Jin, Liang Hong, Yujie Wang, Jie Zhang

**Affiliations:** 1 School of Physics and Astronomy, Shanghai Jiao Tong University, Shanghai, 200240 China; 2 Institute of Natural Sciences, Shanghai Jiao Tong University, Shanghai, 200240 China; 3 School of Material Sciences, Shanghai Jiao Tong University, Shanghai, 200240 China

## Abstract

The boson peak, which represents an excess of vibrational states compared to Debye’s prediction at low frequencies, has been studied extensively, and yet, its nature remains controversial. In this study, we focus on understanding the nature of the boson peak based on the spatial heterogeneity of modulus fluctuations using a simple model system of a highly jammed two-dimensional granular material. Despite the simplicity of our system, we find that the boson peak in our two-dimensional system shows a shape very similar to that of three-dimensional molecular glasses when approaching their boson peak frequencies. Our finding indicates a strong connection between the boson peak and the spatial heterogeneity of shear modulus fluctuations.

## Abstract (web summary)

The low-frequency collective vibrational modes, known as the boson peak, characterize many glasses at low temperature, yet its origin remains elusive. Zhang et al. show a correlation between the boson peak and the spatial heterogeneity of shear modulus fluctuation in a two-dimensional granular system.

## Introduction

Anomalous behaviors of vibrational properties have been found in a broad spectrum of amorphous solids, including inorganic molecular glasses^1–3^, metallic glasses^4^, polymer glasses^5, 6^, colloidal glasses^7, 8^, disordered biological matter^9^, and even vibrated granular glasses^10^, spanning many length scales. These fascinating anomalous behaviors can manifest as valleys in the time correlation of a structure function^11^, and as peaks in either the vibrational density of states (DOS) or the specific heat of Debye’s model^1, 3, 4^. These peaks are referred to as the boson peak (BP) and represent an excess of vibrational states relative to Debye’s prediction at low frequencies^6^, which was first discovered through an experimental study of low-frequency modes in vitreous silica conducted in the mid 1980s^12^. The BP is key to understanding the vibrational properties of amorphous solids and constitutes one of the most challenging problems in the field of condensed matter physics.

Despite the ubiquity of the BP in glassy and amorphous materials, its nature and origins remain under intense debate, for several reasons. First, in experiments using Raman, neutron, and X-ray scattering techniques^1–3^, the essential particle-scale dynamical heterogeneities and spatial heterogeneities of modulus fluctuations are largely missing. To identify microscopic details, a number of experiments have been performed using colloids^7, 8, 13, 14^ and granular particles^10, 15^ to analyze the vibrations of amorphous solids at the single-particle level. However, difficulties can arise when applying the covariance-matrix method^16^, and contact-level stress information is largely missing. Second, some studies have generated controversial results. For example, Ruffle et al. found that the BP corresponds to the Ioffe–Regel limit of longitudinal phonons^17^, while the opposite result was found in other experimental^18, 19^ and numerical studies^20, 21^. In addition, a recent experiment conducted by Chumakov et al. challenges the role of disorders in BP formation^3^. Third, over the past few decades, despite intense effort, various theories have been presented from different perspectives, and as a result, no unified and coherent conclusions have been drawn. Such theoretical works include double-well potential models^22^, soft modes^23^, spatial inhomogeneity of density fluctuations^24^, the soft potential model^25, 26^ and its latest version (the quasi-localized vibration model)^27^, the BP as a signature of the glass transition^28^, the Ioffe–Regel limit^20^, the van Hove singularity^2, 29^, the spatial heterogeneity of the elastic modulus^30, 31^, and the Jamming scenario^32–34^. Given the above complexity, the disagreement among results remains far from being resolved.

Of the theoretical approaches available, one important set of theories is based on the assumption that the BP is strongly connected to spatial fluctuations of the modulus, especially the shear modulus^30, 33, 35^, which was highlighted by Sokolov as being important^36^. Such models^30, 33, 35^ employ a strictly harmonic approach and can be regarded as “Ising models of the BP”. However, we are not aware of any direct experimental studies addressing this important type of models.

Here, we present the first experimental evidence relating to the connection between the BP and the heterogeneity of the shear modulus. Specifically, we address the following key question: How is the BP related to the heterogeneity of the shear modulus and to what extent? We investigate the vibrational properties of a two-dimensional (2D) disordered jammed packing of bidisperse disks at the single-particle scale. Using photo-elastic techniques, we accurately measure particle configurations and contact forces to directly construct a dynamical matrix (or Hessian matrix). Although the system is simple, we find that the BP has a shape similar to that of molecular glasses^1, 27, 37^ around the BP frequency *ω*_b_, despite its markedly different nature in terms of interactions and length scales. Here, we find a strong connection between the BP and the spatial heterogeneity of shear modulus fluctuations (especially for the nonaffine component), supported by the following experimental evidence. First, the transverse (T) wave is strongly dispersive near the BP, while the longitudinal (L) wave is not, which is consistent with studies performed in molecular glasses, suggesting the key role played by shear modulus fluctuations. Second, the phase velocities of the T wave obtained from its dispersion show a minimum near the BP frequency, consistent with effective medium theories^30, 31, 33^. Third, the characteristic length associated with the heterogeneity of the shear modulus (when considering nonaffine contributions) is consistent with the wavelength of the T wave at the BP. Finally, a strong spatial correlation exists between the nonaffine shear modulus and low-frequency modes, supporting the importance of the local nonaffine shear modulus for BP formation.

## Results

### Experimental protocol

**Fig. 1:**
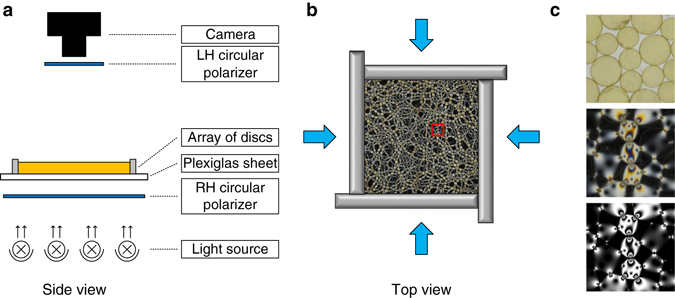
Schematic of the experimental setup. **a** Side view of the setup. **b** Top view of the setup, which includes a square frame filled with ∼1300 bidisperse photo-elastic disks with a number ratio of 1:1 and a size ratio of 1.4:1. The four thick *blue arrows* represent the isotropic compression applied by simultaneously moving the four walls inwards. The small *red square* included in the stress image of a highly jammed packing contains a small portion amplified in **c**. **c** Top: image taken without a polarizer to record the packing’s configuration. Middle: corresponding stress image. Bottom: reconstructed stress image taken from the measured contact forces

We use a biaxial device (biax) to prepare highly jammed packings of bidisperse photo-elastic disks. The biax is mounted on a horizontal Plexiglas plate. A schematic of the setup is shown in Fig. 1. A right-handed circular polarizer is attached under the Plexiglas plate, and a light source is placed ∼30 cm below the polarizer sheet to provide uniform illumination. A high-resolution camera is mounted ∼2 m above the biax to record images of the packings, and a left-handed circular polarizer is placed immediately underneath the camera. A detailed schematic of the arrangement is shown in Fig. 1(a). Further information on the experimental setup is available in the “Methods” section. A sample stress-chain image of a jammed packing is plotted in Fig. 1(b), and a small portion is amplified in Fig. 1(c) to show the particle configurations and their corresponding stress chains. The *bottom panel* of Fig. 1(c) presents a computer-reconstructed stress-chain image based on the contact forces measured between disks to illustrate the reasonably high accuracy of the contact-force measurement approach.

### DOSs and the corresponding reduced DOSs

**Fig. 2:**
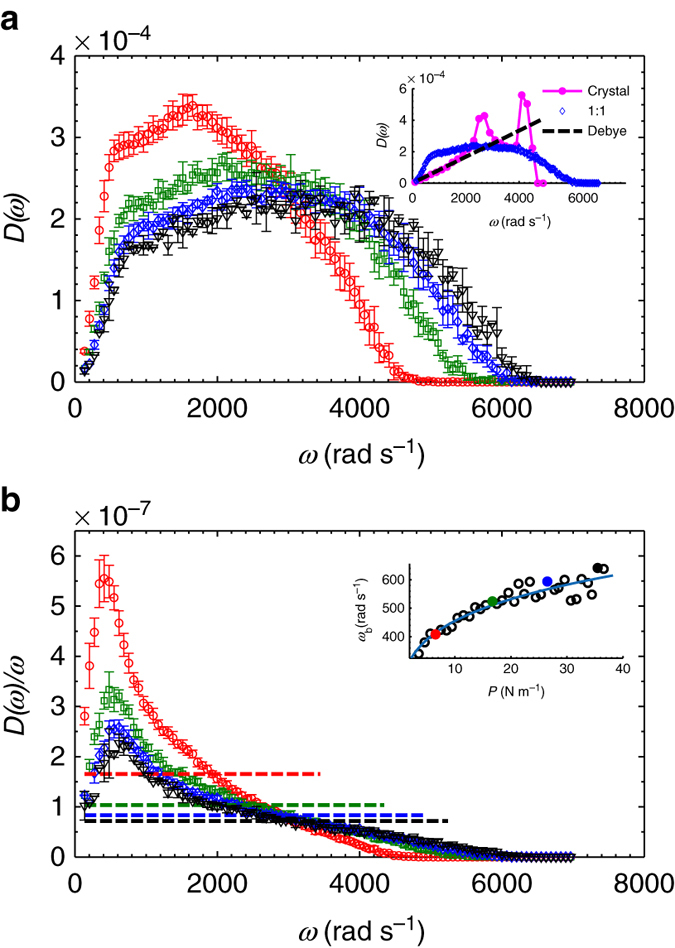
Density of states and the reduced density of states. **a** *D*(*ω*) for pressure (units N m^−1^) *P* = 6.54 (*red circles*), 16.69 (*green squares*), 26.5 (*blue diamonds*) and 35.48 (*black inverted triangles*). Here each curve is ensemble averaged over 10 different runs at the same pressure. Inset: comparisons of *D*(*ω*) values of the disordered system at a pressure of *P* = 26.5 N m^−1^ (*blue diamonds*) with those of the perfect hexagonal lattice (*magenta*) and Debye’s model (*black dashed line*). **b** The corresponding reduced density of states *D*(*ω*)/*ω* after normalizing the *D*(*ω*) in **a** with Debye’s scaling, i.e., *ω* in 2D. Dashed flat lines of the same color denote the corresponding Debye’s model, which is defined as ${g_{\rm{D}}}(\omega )/\omega {\rm{ = }}\frac{S}{{4\pi N}}\left( {\frac{1}{{v_{\rm{L}}^2}} + \frac{1}{{v_{\rm{T}}^2}}} \right){\rm{ = }}\frac{{\rho S}}{{4\pi N}}\left( {\frac{1}{{\left( {B + G} \right)}} + \frac{1}{G}} \right)$, where ${v_{\rm{L}}}{\rm{ = }}\sqrt {\frac{{B + G}}{\rho }}$ is the longitudinal sound velocity, ${v_{\rm{T}}}{\rm{ = }}\sqrt {\frac{G}{\rho }}$ is the transverse sound velocity, *ρ* is the mass density (mass per unit area), *S* is the area of the whole system, *B* is bulk modulus, *G* is shear modulus, and *N* is the total number of particles of the system. Inset: the BP frequency *ω*_b_ vs. the pressure *P*. Here the *blue solid line* is a power-law fitted curve, and the fitting function is *ω*_b_ = 271.5*P* ^0.224^. The four *filled circles* of different colors represent the data points of the four curves of the matching colors shown in the main panel. Here all the error bars denote one SD around the mean value obtained from ∼10 realizations of each pressure

The measured DOSs at pressures of *P* = 6.54 N m^−1^, 16.69 N m^−1^, 26.5 N m^−1^, and 35.48 N m^−1^ are displayed in Fig. 2(a), which shows that each curve becomes broader as *P* increases. The reduced DOS values, *D*(*ω*)/*ω*, are plotted in Fig. 2(b), where each curve of *D*(*ω*) in Fig. 2(a) is normalized over Debye’s scaling (linear with *ω* in 2D), showing BPs in the low-frequency regime. Note multiple definitions of the BP exist^28^: For instance, in our experiment, we can define the BP by comparing the DOSs of amorphous solids with those of corresponding crystals, as shown in the inset of Fig 2(a). Alternatively, we can define the BP by comparing the DOSs of amorphous solids with Debye’s scaling *ω* ^*d*−1^ (dimension *d* = 2 in 2D) and then extracting the peak from *D*(*ω*)/*ω* ^*d*−1^. Here, we adopt the latter approach and use *D*(*ω*)/*ω* ^*d*−1^ to define BP, which is widely adopted in literature^2, 20, 27, 28, 30, 31, 35^. As highlighted in this paper^28^, this approach reveals the universal properties of glasses. Each curve was ensemble averaged over 10 different runs at the same pressure. The height of the BP decreases significantly as the pressure increases, while its *ω*_b_ increases only slightly. The dependence of *ω*_b_ on the pressure *P* is illustrated in the inset of Fig. 2(b), in which the results can be fit using *ω*_b_ = 271.5*P* ^0.224^. The BP regime in Fig. 2(b) is determined from the portion of *D*(*ω*)/*ω* above Debye’s model; flat dashed lines of the same color are defined as ${g_{\rm{D}}}(\omega )/\omega = \frac{S}{{4\pi N}}\left( {\frac{1}{{v_{\rm{L}}^2}} + \frac{1}{{v_{\rm{T}}^2}}} \right) = \frac{{\rho S}}{{4\pi N}}\left( {\frac{1}{{\left( {B + G} \right)}} + \frac{1}{G}} \right)$, where ${v_{\rm{L}}} = \sqrt {\frac{{B + G}}{\rho }}$ is the longitudinal sound velocity, ${v_{\rm{T}}} = \sqrt {\frac{G}{\rho }}$ is the transverse sound velocity, *ρ* is the mass density (mass per unit area), *S* is the area of the whole system, *B* is the bulk modulus, *G* is the shear modulus, and *N* is the total number of particles in the system.

Note that many important characteristics of the BP shown in Fig. 2(b), such as the peak frequency *ω*_b_, peak height *H*_b_, and peak width of the present system (*N* ≈ 1300 particles in total) are nearly identical to those of the system of infinite size (*N*→∞), as obtained by extrapolating a finite size analysis with a maximum difference of ∼1%. In fact, the shape of the BP remains unchanged when *N* ≥ 1245, except at the lowest frequency (results not shown). Below the lowest frequency, which we cannot resolve in the present system, interesting physics processes, such as Rayleigh scaling *ω* ^*d*+1^ (*d* is the dimension, *d* = 2 in 2D and *d* = 3 in three dimension (3D)), may be present, as predicted by the theories of the soft potential model^25, 26^ and the related version (the quasi-localized vibration model)^27^ or through coherent-potential-approximation calculations of heterogeneous elasticity theory^30, 31, 38^. A detailed analysis of the finite size effect lies beyond the scope of our present investigation and will be presented elsewhere (manuscript in preparation).

### Spatial distributions of modes

**Fig. 3:**
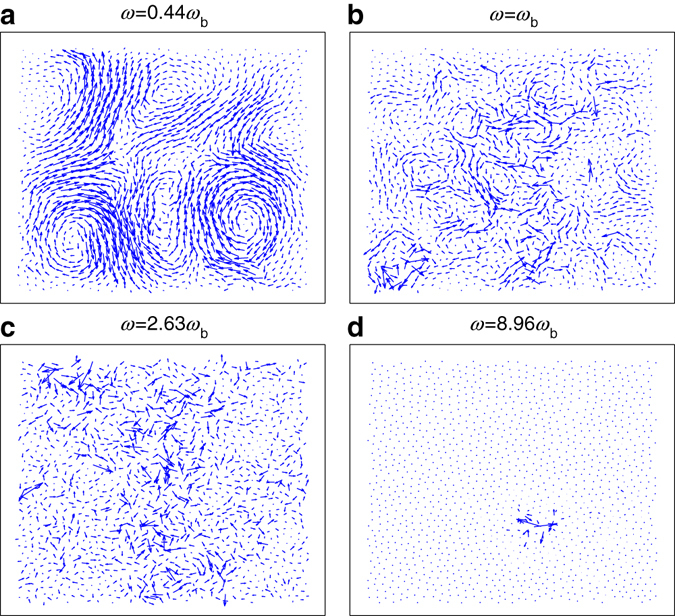
Spatial distributions of modes. The spatial distributions of the modes at *ω*/*ω*_b_ = 0.44 **a**, 1.0 **b**, 2.63 **c**, and 8.96 **d**. The participation ratios are *p*(*ω*_*n*_) = 0.44 **a**, 0.3 **b**, 0.244 **c**, and 0.00499 **d**. Here $p\left( {{\omega _n}} \right) \equiv \frac{{{{\left( {\mathop {\sum}\nolimits_i {m_i}{{\left| {{e_{n,i}}} \right|}^2}} \right)}^2}}}{{\left( {N\mathop {\sum}\nolimits_i m_i^2{{\left| {{e_{n,i}}} \right|}^4}} \right)}}$, where *e*_*n*,*i*_ is the polarization vector of the particle *i* in the *n*th mode (see, e.g., ref. ^8^). Note that the magnitudes of the polarization vectors in different modes are shown at different scales

At different *ω*, the spatial distributions of the polarization vectors differ. Typical results are plotted in Fig. 3(a–d), at *ω* = 0.44*ω*_b_, *ω* = *ω*_b_, *ω* = 2.63*ω*_b_, and *ω* = 8.96*ω*_b_. Here, *ω*_b_ is the BP frequency. Below *ω*_b_, the mode is relatively coherent, displaying collective vibrations at a predominant length scale interwoven with small-scale features, as shown in Fig. 3(a). As *ω* increases, the characteristic length scale decreases, and particles vibrate more randomly, as shown in Fig. 3(b, c). These behaviors are similar to those of colloidal systems^7, 8, 13, 14^. When *ω* ≫ *ω*_b_, a highly localized vibration mode similar to that of refs. ^8, 39^ is observed, as shown in Fig. 3(d). The participation ratio *p*(*ω*_*n*_) (see, e.g., ref. ^8^) of these four modes are as follows: (a) 0.44, (b) 0.3, (c) 0.244, and (d) 0.00499. Similar to colloidal systems in the jammed regime, where the mode is quasi-localized in the vicinity of the BP frequency *ω*_b_, we observe quasi-localized modes for *ω* near *ω*_b_^8^, suggesting the presence of a universal characteristic independent on the particle-level features of individual systems. Note that our system is far from reaching the jamming point. In numerical studies of jamming^40^, the dimensionless pressure *P*_0_ is used to quantitatively characterize how far a system is from reaching the jamming point. The *P*_0_ typically ranges from *P*_0_ = 10^−8^ to *P*_0_ = 10^−3^, and *P*_0_ = 10^−2^ is considered far away from the jamming point. We convert the pressure *P* to the corresponding dimensionless pressure *P*_0_ using the standard procedure described in the literature^40^, and the corresponding results are shown in Table 1. Here, *P*_0_∈[0.155, 0.385], which is orders of magnitude larger than systems near the jamming point. Corresponding values of packing fractions and coordination numbers are also presented in Table 1, where the coordination numbers *Z* are well above the 2D isostatic coordination number *Z*_iso_ = 4 (ref. 40). Additionally, the ratio of the shear modulus to the bulk modulus *G*/*B*≪1 for a system near the jamming point (e.g., *G*/*B* ∼ 10^−4^ when *P*_0_ ∼ 10^−8^, and *G*/*B* → 0 exactly at the jamming point as the system barely gains rigidity)^34, 40^. In contrast, *G*/*B* ≈ $1 \over 3$ in our system, reflecting the same trends for most dense glass-forming systems, where *G*/*B*∈[0.28, 1]; e.g., *G*/*B* ≈ 0.7–1 for vitreous silica and *G*/*B* ≈ 0.3 for OTP (ortho-terphenyl)^41^. In particular, *G*/*B* of our system is close to those of most bulk metallic glasses (*G*/*B*∈[0.17,0.44])^42^.

**Table 1:** Dimensionless numbers of packing

*P* (N m^−1^)	6.54	16.69	26.5	35.48
*P*_0_	0.155	0.257	0.33	0.385
Φ	0.850	0.857	0.862	0.865
*Z*	4.556	4.706	4.729	4.774

The corresponding dimensionless pressure *P*_0_ determined according to the standard procedure described in the literature (see, e.g., ref. ^40^). So the re-scaled pressure *P*_0_ is defined as *P*/(*k*_*n*_ *D* ^*α*−*d*^),where *k*_*n*_ is the normal spring constant, *D* is the average diameter,*α* is the power exponent of the potential vs. deformation, and *d* is the dimension. The last two rows show the values of corresponding packing fractions and coordination numbers

### Comparison between our system and molecular glasses

We can draw quantitative comparisons between our system and the molecular glasses by comparing the ratios of Debye’s frequency *ω*_D_ (i.e., the characteristic frequency of a system) to the BP frequency *ω*_b_. Using data from Buchenau et al.’s^43^ study and the references therein, we compute the $\omega_{\rm D} \over \omega_{\rm b}$ values for the 11 different molecular glasses; the results are listed in Table 2. We find that this ratio falls within a range of $\omega_{\rm D} \over \omega_{\rm b}$ = [4.632, 11.39]. In our system, this ratio $\omega_{\rm D} \over \omega_{\rm b}$ ≈ 8.58 corresponds to the middle of the range. In contrast, for systems that are close to reaching the jamming point, this ratio is orders of magnitude larger and approaches infinity at the jamming point^34^.

**Table 2:** Comparison of *ω*_D_/*ω*_b_

**Substance**	**CKN**	**C**_**2**_ **H**_**5**_ **OH**	**d-SiO**_**2**_	**Se**	**GeO**_**2**_	**PB**
*ω*_D_/*ω*_b_	4.632	5.326	5.705	5.747	6.214	7.446
**Substance**	**Our system**	**B**_**2**_ **O**_**3**_	**a-SiO**_**2**_	**SiO**_**2**_	**PS**	**PMMA**
*ω*_D_/*ω*_b_	8.58	9.429	9.828	9.878	10.43	11.39

Comparison of the specific values of *ω*_D_/*ω*_b_ based on our results and experimental data on molecular glasses collected from ref. ^43^

**Fig. 4:**
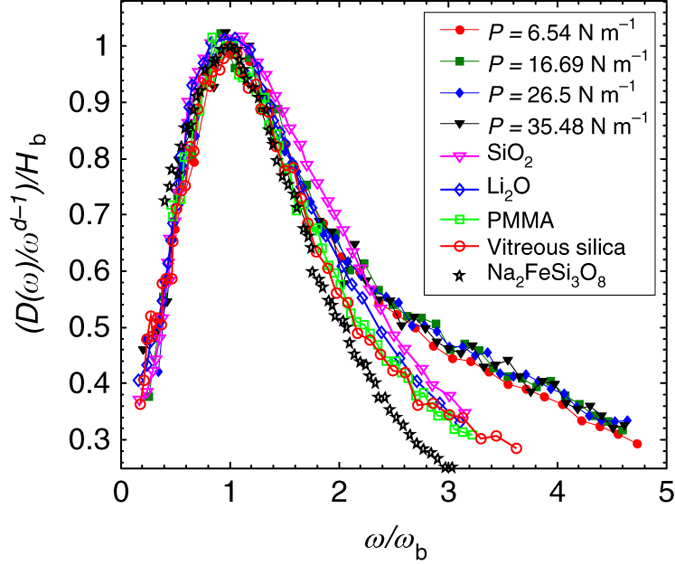
Shape of rescaled BPs. Comparison of the re-scaled BPs ((*D*(*ω*)/*ω* ^*d*−1^)/*H*_b_ vs. *ω*/*ω*_b_) of our results and those found by Buchenau and colleagues^27^ for vitreous silica (*red open circles*) and PMMA (*green open squares*), those found by Sokolov and colleagues^1^ in molecular glasses Li_2_O (*blue diamonds*) and SiO_2_ (*magenta inverted triangles*), and those found by Monaco et al.^37^ in Na_2_FeSi_3_O_8_ glasses (*black stars*). Here *H*_b_ is the height of BP, *ω*_b_ is the peak frequency, and *d* is the dimension with *d* = 2 in 2D and *d* = 3 in 3D. The filled symbols represent the data from the current experiment, and different symbols indicate different pressures corresponding to the curves in Fig. 2(b)

In addition to our comparison of the $\omega_{\rm D} \over \omega_{\rm b}$ values, we also compare the shape of the BP with molecular glass measurements^1, 27, 37^. To this end, we apply Sokolov et al.’s^1^ rescaling method by dividing the vertical axis by the height of the peak *H*_b_ and dividing the horizontal axis by the peak frequency, *ω*_b_. We find that the re-scaled curves exhibit reasonably good collapse results, as shown in Fig. 4. Then, we compare our results with those obtained for the molecular glasses (open symbols)^1, 27, 37^. Interestingly, the shapes are similar, especially near the BP frequency *ω*_b_, as shown in Fig. 4. However, it should be noted that Fig. 4 presents some differences between our results and those obtained for molecular glasses at frequencies that differ substantially from *ω*_b_, particularly at the tails. This deviation can be attributed to a difference in the dimensionality. Indeed, because analytical theories^30, 33, 35^ typically predict a universal shape or scaling behavior of the DOS at the random matrix limit or close to marginal stability states, the normalization of the DOS to Debye’s scaling, which is dependent on dimensionality (linear in 2D and quadratic in 3D), can cause systematic deviations at the low- and high-frequency ends relative to *ω*_b_.

### Dispersion relations of T and L waves

**Fig. 5:**
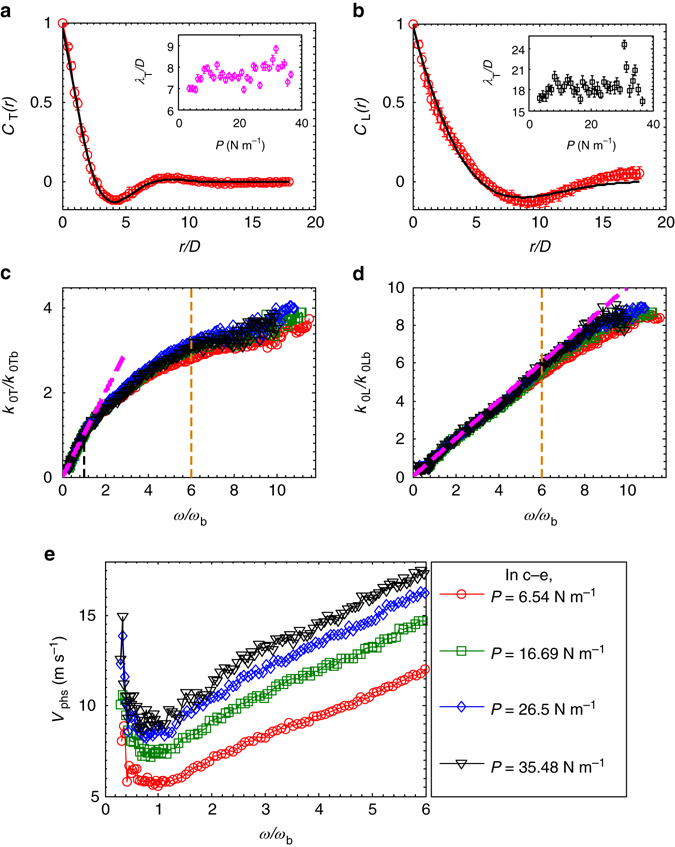
Dispersion relations of T and L waves. **a**, **b** Autocorrelation functions of the polarization vectors: *C*_T,L_(*r*) of the transverse (T) and the longitudinal (L) waves. Here, the pressure *P* = 26.5 N m^−1^, and *C*_T,L_(*r*) is ensemble averaged. The black lines denote fittings based on the function *C*(*r*) = *e* ^−^ $$^{\frac{{{\Gamma _r}}}{2}}$$ cos(*kr* + *ϕ*),where *k*, Γ, and *ϕ* are three fitting parameters. Insets: wavelengths *λ*_T,L_ = $$2\pi \over k_{0T,L}$$ at *ω* = *ω*_b_ vs. pressure. The error bars in **a**, **b** and their insets denote one SD around the mean value obtained from ∼10 realizations of each pressure. **c**, **d** Dispersion relations at pressures of (units N m^−1^) *P* = 6.54 (*red circles*),16.69 (*green squares*), 26.5 (*blue diamonds*), and 35.48 (*black inverted triangles*). Here, $${k_{{\rm{0T,L}}}} = \sqrt {k_{{\rm{T}},{\rm{L}}}^2 + \Gamma _{{\rm{T}},{\rm{L}}}^2/4} $$. The dashed magenta line *k*_0T,L_/*k*_0T,Lb_ = *ω*/*ω*_b_ is used as a guide. The dashed *orange line* at *ω*/*ω*_b_ = 6 represents the starting point of the Anderson localizations, and hence, the dispersion relation is no longer applicable. **e** Phase velocities *V*_phs_ ≡ (*ω*)/(*k*_0T_) of T waves show a prominent dip near BP frequency *ω*_b_ at four corresponding pressures; the actual positions of these dips are at *ω*/*ω*_b_ = 0.960, 0.954, 0.915, and 0.919 from bottom to top. To locate each dip, we use a fourth-order polynomial to reduce the noise and fit the data. Here **c**–**e** are ensemble averaged for ∼10 realizations of each pressure,and the error bars are within the symbol size

After decomposing modes into T waves and L waves following the standard procedure for the *k* space (i.e., the Fourier space^7, 13, 20, 21, 32, 44^), two methods of computing autocorrelation functions are available: The first involves the *k* space, and its resulting autocorrelation functions depend on *k*. The second involves applying an inverse Fourier transform, obtaining separated T waves and L waves in real space, and then performing computations in real space. In real space, we calculate the autocorrelation functions *C*_T,L_(*r*) of the polarization vector of the T waves and L waves at *ω*_b_, as plotted in Fig. 5(a, b). Interestingly, both functions can be well fit using a freely oscillating damped harmonic oscillator *C*(*r*) = *e* ^−^ $$^{\frac{{{\Gamma _r}}}{2}}$$ cos(*kr* + *ϕ*), where $$k = \sqrt {k_0^2 - \frac{{{\Gamma ^2}}}{4}} $$. Here the three fitting parameters are the attenuation coefficient Γ, the characteristic wave number *k*_0_, and the phase angle *ϕ*. The fitted curves are drawn as solid lines in Fig. 5(a, b). Autocorrelation functions *C*_T,L_(*r*) at other frequencies can also be fit using the same function as shown in Supplementary Fig. 1 and discussed in Supplementary Note 1. Note that this fitting function has the similar structure as the forced harmonic oscillator^20, 21, 31^ and the Lorentzian function^13^ described in literature. A characteristic length *λ*_T,L_ = $$2\pi \over k_{0T,L}$$ can be derived from *k*_0_, as plotted in the inset of Fig. 5(a, b).

From *k*_0_, we obtain the dispersion relations of the T waves (a) and L waves (b), as shown in Fig. 5(c, d) for four different values of pressure (see the figure caption for details). Here, the T wave is heavily dispersive around *ω*_b_ while the L wave is not, reflecting the existence of the two modes in the inelastic X-ray measurements of vitreous silica^45^. Using fitting values of *k*_0Tb_ = 68.0 m^−1^ and Γ_Tb_ = 74.54 m^−1^, first, we check that $\frac{{{k_{{\rm{0Tb}}}}}}{{\frac{1}{2}{\Gamma _{{\rm{Tb}}}}}} = 1.82$, consistent with the fitting function *C*(*r*), which has the form of an under-damped harmonic oscillation. Second, the characteristic wavelength $\lambda = \frac{{{\rm{2\pi }}}}{{{k_{{\rm{0Tb}}}}}} = 9.24\,{\rm{cm}}$ and the mean free path $\bar l = \frac{1}{{\frac{1}{2}\Gamma }} = 2.68\,{\rm{cm}}$. Therefore, the Ioffe–Regel limit is not crossed right at *ω*_b_ because $\lambda  >\bar l$, in contrast to findings shown in refs. ^20, 31^. However, some other key aspects of our observations are consistent with the recent numerical studies^31^, such as that the effective shear modulus is strongly frequency dependent, consistent with the changing slope in Fig. 5(c), while the bulk modulus is much less sensitive to the frequency, as indicated by the constant slope in Fig. 5(d). A central prediction of effective medium theories^30, 33, 35^ is that the speed of the T wave, *ν*_phs_ ≡ *ω*/*k*, reaches a minimum value near the BP frequency. We observe this behavior in Fig. 5(e). To find the location of the minimum, we first fit the data with a polynomial to reduce the noise and then find the minimum from the fitting curve. For the four curves shown in Fig. 5(e), we determine that the minimum values are located at $\frac{\omega} {\omega_{\rm b}}$ = 0.960 (*red circles*), $\frac{\omega} {\omega_{\rm b}}$ = 0.954 (*green squares*), $\frac{\omega} {\omega_{\rm b}}$ = 0.915 (*blue diamonds*), and $\frac{\omega} {\omega_{\rm b}}$ = 0.919 (*black inverted triangles*). The average value is $\frac{{\bar \omega }}{{{\omega _{\rm{b}}}}} = 0.937$. According to effective medium theories^31, 33, 46^, the sound velocity of the T wave and the DOS are not independent; instead, they are connected through Kramer–Kronig relations from the Laplace transform of the response function. Hence, the phase velocity of the T wave dips around the BP position *ω*_b_^31, 33, 46^. Our measurement shows that though the dip is not perfectly aligned with the *ω*_b_ (off by 6.3% on average), the experimental results show reasonably good agreement with the predictions of effective medium theories^31, 33, 46^. Interestingly, we also found that Γ and *ϕ* for T and L waves exhibit qualitatively similar behaviors around the BP. However, currently, we do not fully understand the behaviors of Γ and *ϕ* shown in Supplementary Fig. 2.

Note that the results shown in Fig. 5(c–e) depend neither on the means of analyzing data (i.e., whether in real space or in Fourier space), nor on the specific forms of the fitting function. For a self-consistency check, we first compute the autocorrelations in Fourier space, and we then extract *k* and Γ using a Lorentzian fit of the structure functions, as shown in Supplementary Figs. 3 and 4 and discussed in Supplementary Notes 2 and 3. The results of the Fourier space analysis are quantitatively the same as those obtained from the analysis conducted in real space for the regime of $\frac{\omega} {\omega_{\rm b}}$ ≤ 6, as shown in Supplementary Fig. 4(a–d). For $\frac{\omega} {\omega_{\rm b}}$ > 6, results from these two analyses start to deviate significantly from one another as is shown in Supplementary Fig. 4, as autocorrelation functions fail to capture the new physics of this regime. When $\frac{\omega} {\omega_{\rm b}}$ ≈ 6, the spatial distribution of a mode starts transition to an Anderson localization regime in which the statistics become dominated by Poisson statistics rather than by Gaussian orthogonal ensemble statistics^47^. Our detailed analysis of Anderson localization will be presented elsewhere (manuscript in preparation). Hence when $\frac{\omega} {\omega_{\rm b}}$ > 6, as denoted by the vertical dashed line in Fig. 5(c, d) and in Supplementary Fig. 4(a–d), the results obtained from the analyses conducted in real and Fourier space are no longer useful.

### Connection between local-modulus heterogeneity and BP

**Fig. 6:**
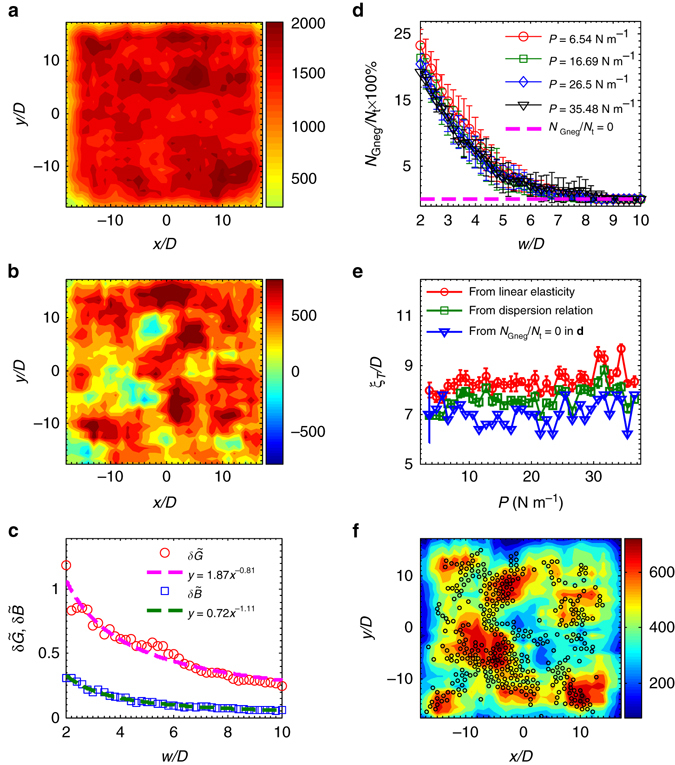
Heterogeneity of local moduli. Contour plot of the local bulk *B* **a** and shear *G* moduli **b** at *P* = 26.50 N m^−1^. Here, the coarse-graining size *w* = 5*D*, where *D* is the mean diameter of the particles. Both *B* and *G* include affine and nonaffine components, and there are negative values in *G* but not in *B*. **c** Relative fluctuations of moduli $\delta \tilde G \equiv \frac{{\delta G}}{{\langle G\rangle }}$ (*red circles*) and $\delta \tilde B \equiv \frac{{\delta B}}{{\langle B\rangle }}$ (*blue squares*) that satisfy the power-law fitting (*dashed lines*). Here, $\delta \tilde B \propto {w^{ - 1.11}}$ and $\delta \tilde G \propto {w^{ - 0.81}}$. The data points here are ensemble averaged for seven realizations of *P* = 26.5 N m^−1^,other pressures show the similar results, and the error bars are within the symbol size. **d** The percentage of the negative *G* vs. *w* at *P* = 6.54 (*red circles*), 16.69 (*green squares*), 26.5 (*blue diamonds*), and 35.48 (*black inverted triangles*) N m^−1^. The characteristic length can be determined from the crossover between each curve and the zero line (i.e., the *magenta dashed line*). **e** Comparison of three characteristic lengths: (1) from linear elasticity values of ${v_{\rm{T}}} = \sqrt {G/\rho }$ and *ξ*_T_ = 2*πv*_T_/*ω*_b_ (*red circles*), (2) from the dispersion relation (*green squares*), and (3) from the above crossover (*blue inverted triangles*). The error bars in **d**–**e** signify one SD around the mean value obtained from ∼10 realizations of each pressure. **f** The spatial correlation between the nonaffine *G* (backgrounds) and the superposition of low frequency modes for *ω* < *ω*_b_ (*black circles*). Here, *w* = 7*D*

A natural question arises from the observations of Fig. 5 described above: Why is the T wave strongly dispersive near the BP, in contrast to the L wave? The results shown in Fig. 5 reveal a qualitative difference between the shear modulus and the bulk modulus. To address this question, we calculate the spatial distributions of the local shear modulus *G* and bulk modulus *B* following a standard approach based on the dynamical matrix^48^. Relevant details can be found in Supplementary Note 4. The corresponding results are shown in Fig. 6(a, b). Note that *G* and *B* include the contributions from both the affine and the nonaffine parts because in amorphous solids, the nonaffine motion is intrinsic^49^. Comparing Fig. 6(a) and (b) reveals a qualitative difference between the spatial distributions of *B* and *G*: there are negative regimes in the distribution of *G*, as shown in panel (b), while a similar behavior is not observed in *B*, as shown in panel (a). Note that a negative regime of the local *G* denotes the existence of a potentially soft regime in the system. Additionally, the above observations were obtained through recent numerical studies^50, 51^, in which a negative regime was found in the spatial fluctuations of *G* but not in those of *B*. In contrast, no negative regimes in *G* would be observed if only the affine part were computed, as shown in Supplementary Fig. 5. Moreover, the averaged relative fluctuation $\delta \tilde G$ is much larger than that of $\delta \tilde B$, indicating the important role of $\delta \tilde G$ in the formation of the BP. Indeed, $\delta \tilde G$ and $\delta \tilde B$ vs. the coarse-graining length *w* satisfy the power law fitting similar to refs. ^50, 51^, as shown in Fig. 6(c). Quantitatively, $\delta \tilde G \propto {w^{ - 0.81}}$ and $\delta \tilde B \propto {w^{ - 1.11}}$ in Fig. 6(c), whereas both $\delta \tilde G \propto {w^{ - 1.02}}$ and $\delta \tilde B \propto {w^{ - 1.13}}$ as shown in Supplementary Fig. 5. These findings are consistent with refs. ^50, 51^. Here the *w* ^−1^ scaling can be understood based on the central limit theorem and the lack of long-range correlations in the spatial fluctuations of moduli, equivalent to Gaussian statistics of the modulus flucutation^31, 50, 51^. In a recent experiment, Wagner et al. used a novel atomic force acoustic microscopy method to measure the distribution of local elastic constants along the surface of metallic glass^52^. Their results revealed that the distribution of a local indentation modulus, which is a combination of both bulk and shear moduli, obeys a Gaussian distribution with must larger fluctuations than those of the crystal^52^. The Gaussian distribution derived from Wagner et al.’s experiment^52^ is consistent with the results shown in Fig. 6(c) and Supplementary Fig. 5, revealing universal Gaussian statistics of local modulus fluctuations in 2D and 3D glassy systems that are independent of dimensions and detailed particle-scale interactions.

A natural length scale can be derived in which a negative regime first appears in the spatial distribution of *G* as the coarse-graining size *w* decreases. Figure 6(d) plots the percentage of the negative *G* as a function of *w* at different pressure levels. The first negative modulus appears at ~*w* = 7*D* for all pressure levels, and as *w* decreases, the percentage increases rapidly. Figure 6(e) compares three characteristic lengths: (1) the length determined from linear elasticity ${V_{\rm{T}}} = \sqrt {G/\rho }$ and *ξ*_T_ = 2*πV*_T_/*ω*_b_ (*red circles*), (2) the length measured from *k*_0_ at *ω*/*ω*_b_ = 1 (*green squares*), and (3) the length obtained from *G* as discussed above (*blue inverted triangles*). Within the measurement uncertainties, these three length scales are very similar. Thus, BP formation is indeed related to fluctuations of the local shear modulus, whereas fluctuations of the bulk modulus show no signs of a characteristic length over all frequencies, consistent with early propositions posed in the literature^31^. Moreover, this result confirms the essential role played by the nonaffine component of fluctuations of the local shear modulus. Finally, we present more supporting evidence in Fig. 6(f), where a spatial correlation between the nonaffine shear modulus (the background) and low-frequency modes (*black circles*) is clearly evident. Here, *w* = 7*D*, equal to the length scale derived from Fig. 6(e). Hence, the nonaffine component of the shear modulus plays a central role in BP formation and is related to potential soft regimes in the system. Indeed, previous studies have alluded to a direct connection between low-frequency modes and “soft spots”, i.e., locations where local plastic rearrangement tends to occur under conditions of external perturbation^8, 53–55^.

The affine and nonaffine moduli discussed above are consistent with the terminologies used by the metallic glass community to describe the elastic heterogeneity of metallic glasses (e.g., the binary mixture of liquid-like and solid-like atoms^56, 57^, the core–shell model^58^, the mixture of flow units and elastic matrix^59^, and the mixture of strongly bonded and weakly bonded regions^60^). These names carry similar physical meanings to facilitate theoretical modeling. However, they do not denote a clear boundary between two different regimes, such as between solid- and liquid-like atoms or between affine and nonaffine moduli. Demowski et al.^56^ found that a ∼75% volume fraction of metallic glasses deforms elastically (affinely), whereas the rest deforms nonaffinely without resistance. They also determined that the ratio between nonaffine and affine Youngs moduli is ~24–28%. We estimate a ratio ∼34% based on our experiment, consistent with the results reported by Demowski et al.^56^ Moreover, our finding that the BP and nonaffine shear modulus are strongly related is qualitatively consistent with the recent experimental work reported by Luo et al.^4^, who found a link between the fast dynamics of the BP and the slow dynamics of structural relaxation in metallic glasses. We believe that the slow dynamics of structure relaxation affect the nonaffine shear modulus to a great extent and, in turn, influence the properties of the BP^4^.

## Discussion

In conclusion, using a highly jammed 2D granular material as a model system, we study the vibrational modes of an amorphous system, focusing on the relationship between the BP and the spatial heterogeneity of modulus fluctuations. Our main finding is that BP formation is closely related to the spatial heterogeneities of shear modulus fluctuations and, in particular, to the nonaffine component of these fluctuations. This strong relationship manifests in several aspects of vibration modes. First, the qualitative difference between the dispersion relations of T waves and L waves suggests that the spatial fluctuations of the shear modulus play a critical role in BP formation; indeed, there is a strong dispersion of T waves near the BP frequency, while L waves are linearly dispersive. Second, the phase velocity of T waves obtained from the dispersion relation shows a dip close to the BP frequency, confirming the predictions of effective medium theories^30, 31, 33, 35, 46^, which assume that spatial fluctuations of the shear modulus are central to BP formation. Third, our modulus fluctuation measurements confirm that the relative fluctuations of the shear modulus are much greater than those of the bulk modulus. Thus, a characteristic length scale can be estimated from the spatial fluctuations of the shear modulus that is comparable to the wavelengths of T waves at the BP frequency. Finally, the spatial correlation between the nonaffine shear modulus and low-frequency modes demonstrates the importance of the nonaffine shear modulus for BP formation.

The molecular structures of real molecular glasses can be rather complex, potentially introducing certain features beyond those that our model system—a strictly harmonic system—can fully capture. For example, vitreous silica^12, 61^, for which the BP is related to local structure relaxation due to polyamorphic transformation processes (i.e., coupled tetrahedral libration), requires the inclusion of anharmonicity terms in the soft potential and in related models^25–27^ to restore system stability. Nonetheless, our description and analysis of the BP reveals salient features that have also been observed in vitreous silica^12, 25–27, 61^ (e.g., the collective movements of particles around the BP frequency). In addition, our analysis of the nonaffine shear modulus and its relationships to the BP essentially highlights the collective movements of particles to restore the system to mechanical equilibrium as a result of disorder in which affine motion alone cause particles to become unstable. This approach is essentially the same as those used in the soft potential and related models^25–27^, indicating that collective soft modes are responsible for BP formation.

## Methods

### Experimental details

In this experiment, we used a state-of-the-art biaxial apparatus to create 2D amorphous solid packing at different pressure levels. This apparatus mainly consists of a rectangular frame mounted on top of a transparent and powder-lubricated glass plate with four walls that can move symmetrically while keeping the center of mass fixed, unlike those reported in refs. ^62–65^. The rectangular area was filled with a random mixture of ∼1300 bidisperse photo-elastic disks of with diameters of 1.4 cm and 1.0 cm and a number ratio of 1:1 (reflecting the jamming protocol^32, 40^), to create various random initial configurations slightly below the jamming point. Next, we applied isotropic compression to achieve packing at particular pressure levels and performed 10 different runs following the same protocol to create an ensemble. In each run, we recorded images of the particle configurations and the packing stress information. We then employed image processing and a force-inverse algorithm to extract geometric information from each particle and the contacts and contact forces between particles. In preparing the jammed packing, we constantly applied mechanical vibrations before the system’s packing fraction exceeded ∼84% (i.e., the typical isostatic jamming point)^32, 40^. When using this approach, the prepared packing was essentially the same as that of a frictionless packing, and the typical contribution to the total elastic energy level due to tangential forces was two orders of magnitude less significant than that of normal contact components. Plotting the pre-calibrated curve of the contact forces (i.e., the normal and tangential components) vs. the deformation, we determined the spring constants at each contact point to obtain a complete set of data for constructing the system’s Hessian matrix. These results will be reported elsewhere (manuscript in preparation).

### Data availability

The data that support the findings of this study are available from the corresponding author upon request.

## Electronic supplementary material

Supplementary Information
