# Supplementary material for: Experimental studies of vibrational modes in a two-dimensional amorphous solid
Source: Nat Commun. 2017 Jul 10;8:67. doi: 10.1038/s41467-017-00106-5 (PMC5503991; doi:10.1038/s41467-017-00106-5)
Supplement: Supplementary file 1 — Supplementary Information [file 41467_2017_106_MOESM1_ESM.pdf]

File name: Supplementary Information
Description: Supplementary figures, supplementary notes and supplementary references.

Supplementary Information

Supplementary Note 1: Autocorrelation functions of T and L waves

For a given pressure, the autocorrelation function within the regime of the BP can be fit well using the form of a freely oscillating damped harmonic oscillator $C(r) = e^{-\frac{\Gamma r}{2}} cos(kr+\phi)$, where $k = \sqrt{k_0^2 - \Gamma^2/4}$, with three fitting parameters (i.e., the damping coefficient Γ, the intrinsic wave number k_0, and the phase constant ϕ). Here, these panels show the autocorrelations of T and L waves for three representative frequencies: $\omega = 0.36\omega_{\mathrm{b}}$ in Supplementary Figure 1(a-b), $\omega = \omega_{\mathrm{b}}$ in Supplementary Figure 1(c-d), and $\omega = 4.64\omega_{\mathrm{b}}$ in Supplementary Figure 1(e-f). Here, the pressure is $\sim 26.5\mathrm{Nm}^{-1}$, and results are similar to those of other pressures. Black lines are fitting curves.

Supplementary Note 2: Analyses in Fourier Space

The results of dispersion relations and Γ-versus-ω relations are independent of the actual way of analyzing the data, either in real space or in Fourier space. We also analyzed modes in Fourier space. First, we decomposed a mode into the transverse and longitudinal part in Fourier space based on the fact that for transverse waves components are perpendicular to wave vectors, whereas for longitudinal waves components are parallel to wave vectors[1]. Results are shown in Supplementary Figure 3(a-b) at $\omega = \omega_{\mathrm{b}}$, and these results are similar to those of other frequencies. Structure factors ($s_{\mathrm{T}}(k,\omega)$ or $s_{\mathrm{L}}(k,\omega)$) can be readily computed, as shown in panels(c-d). In Supplementary Figure 3(c-d), solid lines represent the Lorentzian fitting (see figure captions for details).

Supplementary Note 3: Real-space and Fourier-space analyses

Here is the comparison between real-space analyses (red circles) and Fourier-space analyses (dark blue squares) for dispersion relations and Γ versus ω at $P = 26.5\text{Nm}^{-1}$, as shown in Supplementary Figure 4(a-d). Magenta dashed lines are guide to eyes. The orange dashed lines at $\omega/\omega_\text{b} = 6$ denote the starting point of the Anderson localization, beyond which the results of both analyses are no longer meaningful. Black dash lines indicate BP positions, in all panels, the horizontal and vertical axes are re-scaled by the corresponding BP frequencies. So from this figure, we see that the dispersion relations obtained by both methods are quantitatively the same within $\omega \leq 6\omega_\text{b}$, independent of specific means of analysis.

Supplementary Note 4: Computation of local bulk and shear moduli

To determine the local shear and bulk moduli centered on a given particle within a square domain of box size w/D with D the average particle diameter, we applied a method introduced in the paper[2]. We find that it makes very little difference whether the selected domain is a square or a circle. We first applied a uniform and infinitesimal virtual compression (or pure-shear) strain field to create a displacement field of the individual disks within the box. Therefore, a disk i would have a displacement u_i subjected to this strain: in isotropic compression, displacements are $u_i = \gamma r_{ci}$, whereas in pure shear, displacements are $u_{ix} = \gamma x_{ci}$ and $u_{iy} = -\gamma y_{ci}$. Here, r_{ci} are position vectors with respect to the center of the box, and x_{ci} and y_{ci} are their corresponding horizontal and vertical components. From individual displacement vectors, relative deformation between a pair of contacted disks i and j was derived for the normal component $u_{\parallel,ij}$ and the tangential component $u_{\perp,ij}$: $u_{\parallel,ij} = u_{ijx} cos\theta_{ij} + u_{ijy} sin\theta_{ij}$, $u_{\perp,ij} = u_{ijy} cos\theta_{ij} - u_{ijx} sin\theta_{ij}$, where the θ_{ij} represents an angle between the position vector pointing from i to j and the horizontal axis. Second, from the linear response, the change of contact forces was then computed directly. Third, combining information of contact forces and contact positions allowed us to compute the stress tensor σ_{ij} of this given box to derive B and G using $B = \frac{\sigma_{xx}+\sigma_{yy}}{4\gamma}$ and $G = \frac{\sigma_{xx}-\sigma_{yy}}{4\gamma}$. Finally, applying the above method to the different locations of the system, we computed all local moduli, as shown in Supplementary

Figure 5, where panel (a) plots the spatial distributions of the local bulk modulus B and panel (b) plots the spatial distributions of the local shear modulus G. For the convenience of plotting, we typically first mapped the system onto a square grid and then calculated local moduli for each grid. Note that the calculation of the local modulus does not depend on the specific treatment of boundary particles of the box. We applied two schemes, and results are insensitive to actual schemes used. The first scheme is a binary one: if the center of a particle is within the box, the particle is included, otherwise, it is excluded. The second scheme is a continuous one: each boundary particle is assigned a numerical weight, which equals the fraction of the disk area within the box over the total disk area.

One important parameter in the above calculation is the box size w/D. To check the dependence of local moduli on w/D, we divided the system into nine non-overlapping regimes and we then chose the centers of these nine regimes to compute the fluctuations of local shear-and-bulk moduli by systematically varying w/D. Results are shown in Supplementary Figure 5(c), where both normalized fluctuations of K and G satisfy a power-law scaling with an exponent close to -1. These results are quite surprising, indicating the absence of characteristic length scales in modulus fluctuations at least for affine components, which is similar to those of a numerical study of systems near the jamming point[3].

To measure the modulus including non-affine components, we used the method in Ref.[4]. Essentially, we first calculated the Hessian matrix $H_{i\alpha j\beta}$, which is constructed from spring constants of all contacts. Then, affine forces $\Xi_{i\alpha}$ were computed using $\Xi_{i\alpha} = H_{i\alpha j\beta}\frac{\partial r_{j\beta}}{\partial\gamma}$,where $r_{j\beta}$ are affine displacement fields subjected to a compressional strain or pure-shear strain γ, and nonaffine displacement fields were obtained using the equation (A4) in the appendix of Ref.[4]. Finally, the non-affine contributions of moduli were calculated using the similar way as the affine parts $B_{\mathrm{nonaffine}} = \frac{\sigma_{xx}^{\mathrm{nonaffine}}+\sigma_{yy}^{\mathrm{nonaffine}}}{4\gamma}$ and $G_{\mathrm{nonaffine}} = \frac{\sigma_{xx}^{\mathrm{nonaffine}}-\sigma_{yy}^{\mathrm{nonaffine}}}{4\gamma}$, and total moduli are the results of affine terms subtracted by the corresponding non-affine terms.

Supplementary References

1. H. Shintani and H. Tanaka. Universal link between the boson peak and transverse phonons in glass. *Nat. Mater.*, 7(11):870–7, 2008.

2. W. G. Ellenbroek, M. van Hecke, and W. van Saarloos. Jammed frictionless disks: Connecting local and global response. *Phys. Rev. E*, 80(6 Pt 1):061307, 2009.

3. H. Mizuno, L. E. Silbert, and M. Sperl. Spatial distributions of local elastic moduli near the jamming transition. *Phys. Rev. Lett.*, 116(6):068302, 2016.

4. C. E. Maloney and A. Lemaitre. Amorphous systems in athermal, quasistatic shear. *Phys. Rev. E*, 74(1 Pt 2):016118, 2006.

5. D Kaya, N. L. Green, C. E. Maloney, and M. F. Islam. Normal modes and density of states of disordered colloidal solids. *Science*, 329(5992):656–658, 2010.

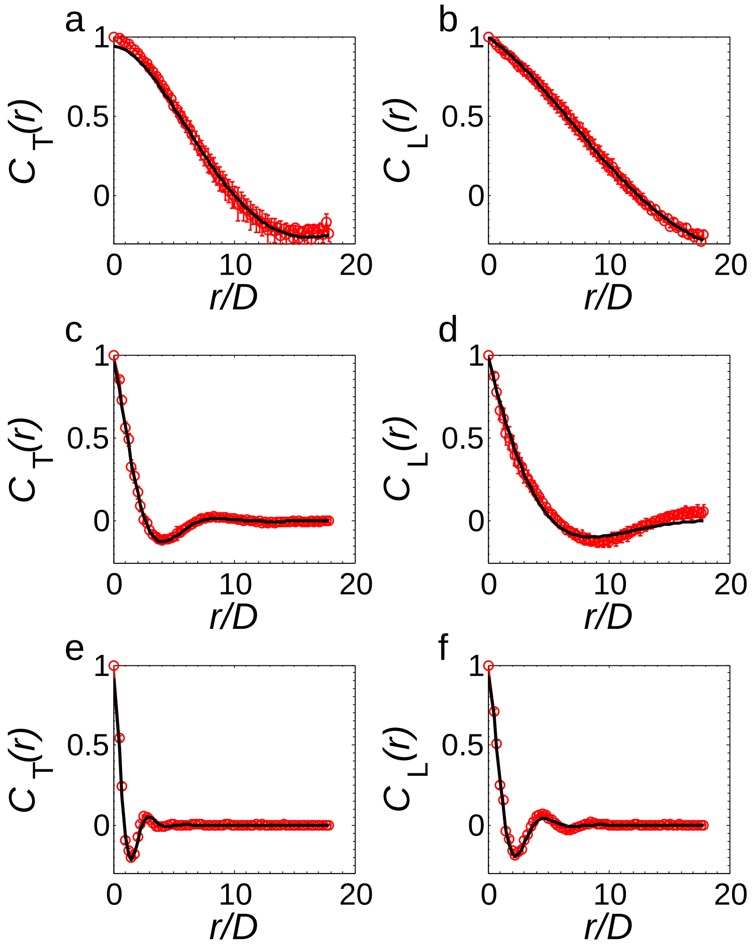

Supplementary Figure 1: Autocorrelation functions $C_{\mathrm{T,L}}(r)$. Autocorrelation functions $C_{\mathrm{T,L}}(r)$ at $\omega = 0.36\omega_{\mathrm{b}}$ (a-b), $\omega = \omega_{\mathrm{b}}$ (c-d), and $\omega = 4.64\omega_{\mathrm{b}}$ (e-f). Here, $P = 26.5\mathrm{Nm}^{-1}$, and results at other pressures are similar. In all panels, black solid lines are the fitting curves using $C(r) = e^{-\frac{\Gamma r}{2}} cos(kr + \phi)$ of a freely-damped harmonic oscillation. In each panel, results were ensemble averaged over 7 different realizations. Here, error bars denote one s.d. around the corresponding mean values of 7 realizations.

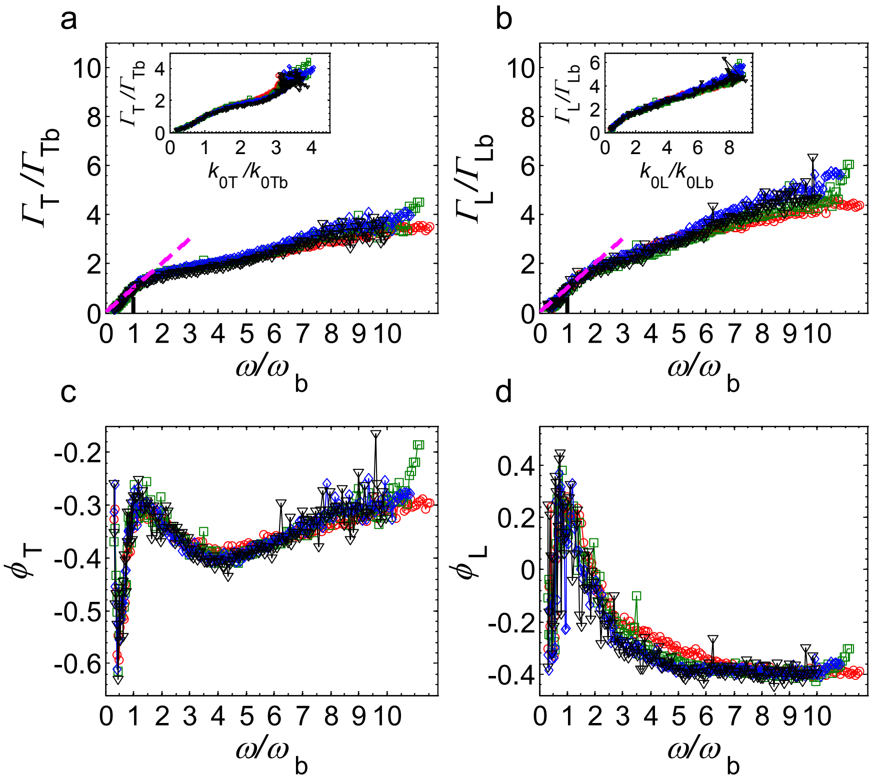

Supplementary Figure 2: Fitting parameters Γ and ϕ versus ω/ω_b. Fitting parameters Γ, and ϕ versus ω/ω_b at the pressure levels of $P = 6.54$ (red circles), 16.69 (green squares), 26.5 (blue diamonds), and 35.48 (black inverted triangles) Nm^{-1}. Panels (a-b): Γ versus ω. Magenta dashed lines are guide to eyes and black dotted lines mark $\omega/\omega_b = 1$. Insets: Γ versus k_0 for T (a) and L (b) waves. Panels (c-d) plot $\phi_{T,L}$ versus ω/ω_b, where both ϕ_T and ϕ_L show the existence of a prominent peak around $\omega/\omega_b = 1$. Here, results were ensemble averaged over ~ 10 realizations of each pressure, and error bars are within the symbol size.

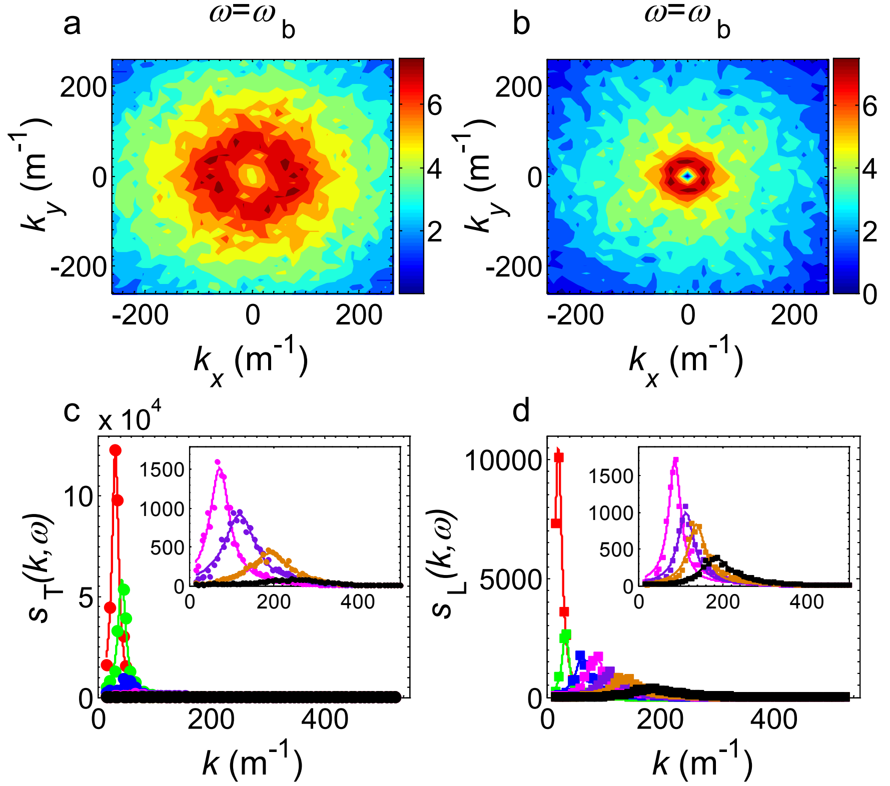

Supplementary Figure 3: T and L waves analysed in Fourier space. Contour plots of the T (a) and L (b) modes at the BP frequency $\omega_{\rm b}$ in Fourier space. Panels (c-d) are the structure factors $s_{\rm T}(k,\omega)$ and $s_{\rm L}(k,\omega)$ of T and L waves versus the wave number k at 7 frequencies. The frequencies of $s_{\rm T}(k,\omega)$ curves are (units $\omega_{\rm b}$) $\omega = 0.50$ (red), 0.62 (green), 0.74 (blue), 1.00 (magenta), 2.06 (purple), 4.04 (orange), and 6.31 (black); The frequencies of $s_{\rm L}(k,\omega)$ curves are (units $\omega_{\rm b}$) $\omega = 0.74$ (red), 1 (green), 2.06 (blue), 2.98 (magenta), 4.04 (purple), 5.04 (orange), and 6.31 (black). Insets in both panels are magnified view of the last four largest frequencies. Here, solid lines represent the Lorentzian fitting $s = c + \frac{2/\pi A\Gamma}{4(k-k_0)^2+\Gamma^2}$ (see e.g. Ref.[5]), where $c \approx 0$ is a small correction constant, A is a constant coefficient, k_0 is the peak position, and Γ is full width at half maximum. Here, results were ensemble averaged over 7 realizations of $P = 26.5{\rm Nm}^{-1}$, and error bars are within the symbol size.

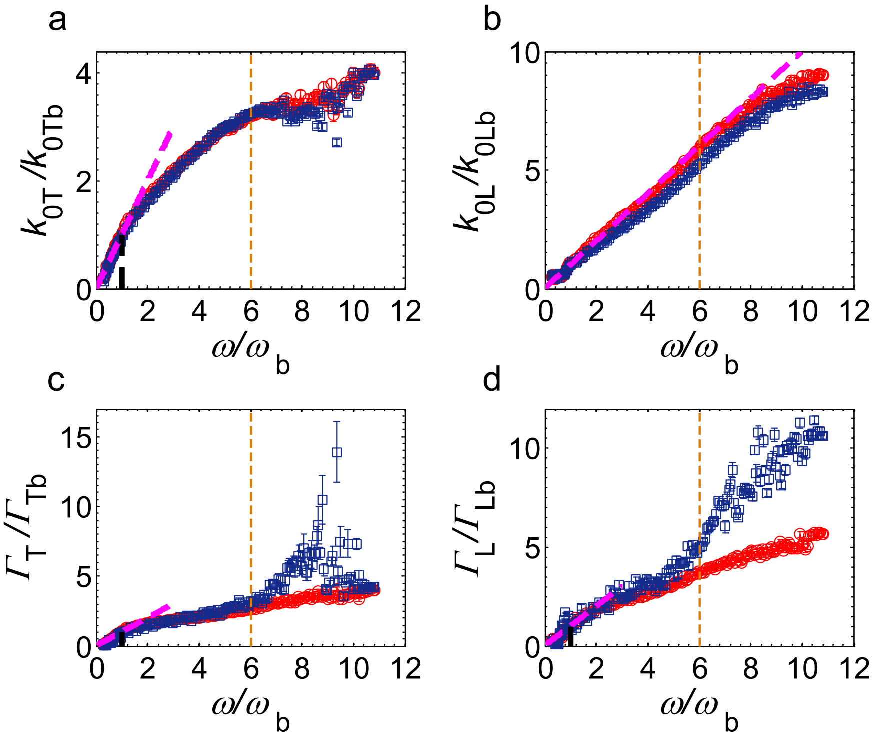

Supplementary Figure 4: Real-space and Fourier-space analyses. The comparison of dispersion relations (a-b) and fitting parameters Γ (c-d) obtained from self-correlations (red circles) in real space and structure factors (dark blue squares) in Fourier space at the pressure $26.5\mathrm{Nm}^{-1}$. Magenta dashed lines are guide to eyes. Orange dashed lines at $\omega/\omega_\mathrm{b} = 6$ represent the start of the Anderson localization, beyond which results are no longer meaningful. Here, results were ensemble averaged over 7 realizations, and error bars are within the symbol size.

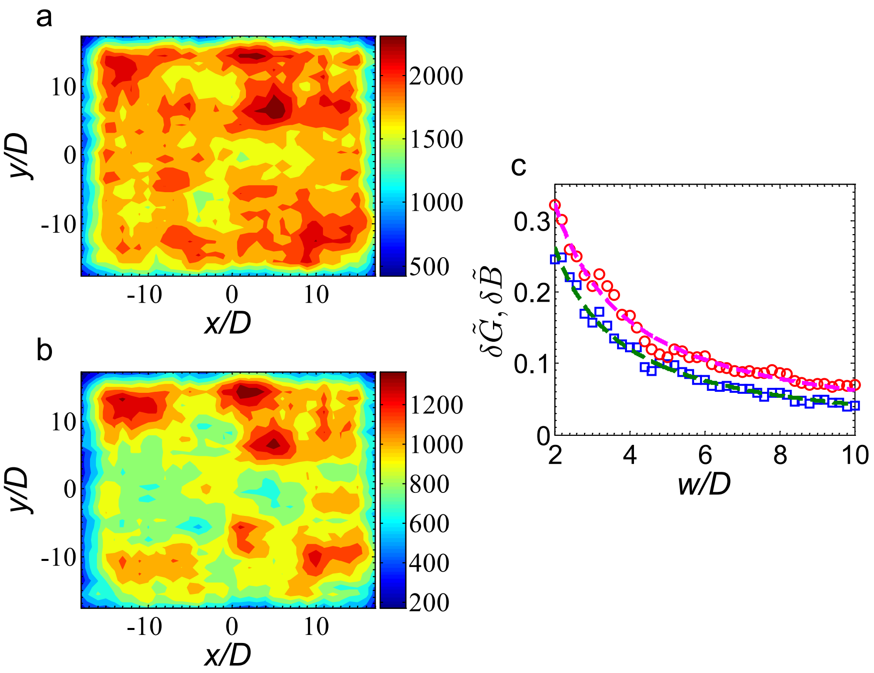

Supplementary Figure 5: Spatial distributions of affine local bulk and shear moduli. Contour plots of the local bulk B (a) and the shear modulus G (b) at the pressure $P = 26.50\text{Nm}^{-1}$ with the box size $w/D = 5$, in which D is the mean particle diameter. Here B and G only include affine components. (c) Relative fluctuations $\delta\tilde{G}$ (red circles) and $\delta\tilde{B}$ (blue squares) versus w/D. To avoid boundary effects and overlaps between domains, each realization was divided into 3×3 boxes, and results were averaged over 7 realizations at the same pressure. Here $\delta\tilde{B} \propto w^{-1.13}$ and $\delta\tilde{G} \propto w^{-1.02}$, showing no clear qualitative differences and no characteristic length scales.
